# Report of Nine Cases of Poisoning by Arsenious Acid

**Published:** 1854-04

**Authors:** R. A. Lewis

**Affiliations:** Richmond, Va.


Report of Nine Cases of Poisoning by Arsenious Acid.—By R. A. Lewis, M. D., Richmond, Va.—On the 15th of February, 1854, at half past seven o'clock, A. M., I was called to see a family, consisting of nine persons, six adults and three children under ten years of age. Six of the nine were white persons, and three were black. Five of the whites were adults, and one a little girl about five or six years old. One of the blacks was an adult; the other two were children—one eight and the other four years old. All the white persons were females except one—a man about twenty-five years of age. All the blacks were females—the adult a woman about forty years old.

I found these nine persons all laboring under the symptoms of poisoning by some irritant poison. The symptoms were violent, nausea and vomiting, faintness, a sense of constriction and burning in the throat, great thirst, pain in the stomach and bowels, quick, irregular pulse, and skin rather cooler than natural.

Upon inquiry, I found that there had been a difficulty with the cook, a black woman, about forty, and she had been notified that she would be sent to the public hiring on that day; and she had replied that she would die before she would go.

The whole white family sat down to breakfast, and in a short time some of them became deadly sick, and began to vomit; and in a short time all who had eaten were vomiting; or rather, all who had drank coffee. The cook was called into the house and accused of putting poison in the coffee, which she denied. She was then told, if she had not done so, to drink some of the coffee. She said she was not afraid to do so, and poured out and drank a cupful, and gave some to each of her children.

I poured the liquid out of the coffee-pot in a bowl, and spread the grounds out in a plate, and found white particles through the whole of them. Placing some of them on my watch, I found it was not corrosive sublimate. As arsenious acid is more commonly used as a poison than any other substance, I commenced my treatment upon the supposition that this was the poison used. I immediately sent for hydrated peroxide of iron; or if that could not be had freshly prepared, magnesia ust. While the messenger was gone, I gave my patients freely large draughts of flour and milk, which was ejected almost as soon as swallowed; but was repeated every few minutes, till the messenger to the druggist returned with the magnesia, the hydrated peroxide of iron not being readily prepared. While administering this in large doses, not being weighed or measured, Dr. Power, who had been sent for, came in, and concurring perfectly with me, we continued to give them the magnesia, and the mucilage of sassafras pith for a drink, (which was given *ad libitum*) till about 2 o'clock, P. M. The vomiting still continuing, large quantities of bile, mixed with food, was ejected, mixed with the magnesia and mucilage, and an occasional tinge of blood, and an action on the bowels occasionally in some of the cases.

About 2 o'clock, P. M., we gave each patient a full dose of laudanum in a little olive oil, and repeated it until it was retained. Mustard plasters over the stomach were also applied. The vomiting gradually ceased, the intervals between the paroxysms becoming longer and longer until 10 o'clock at night—most of them dosing—when we left them.

February 16th.—We were told that our patients rested some through the night, occasionally suffering from nausea, and some of them vomiting several times. We found them all complaining of headache, soreness and tenderness over the stomach, great thirst, skin rather warm, and pulse quick and tense in all the cases, except the black woman, who had been purged most. Her skin was cooler and pulse weaker than the rest. Other symptoms the same. Directed ice to be eaten freely, and a tablespoonful of ice cream three times a day.

17th.—Patients all better. Treatment and diet the same.

18th.—Patients still better; ask for food; small quantity of mush and milk three times a day, and ice as before.

19th.—Patients all sitting up, but weak, asking for meat, bread, &c., but mush and milk diet was prescribed for a week, and the patients dismissed as convalescent.

I have been informed that the black woman, on the 20th, repeated the dose, from which she died. I was not called to her in the second attack, but on the 19th she was as well as the others, all of whom have recovered. Before her death she confessed that she put a white powder in the coffee.

I took some of the decoction of coffee and some of the grounds home with me, and analyzed them, which gave the following result:—

1st.—The acids and zinc to be used were tested and found to contain no trace of arsenic.

2d.—Some of the grounds were carefully dried and heated in a test tube, with pulverized charcoal, which gave the characteristic metalic ring of metalic arsenic.

3d.—The hydrogen, or Marsh's test, was used, and after the coffee was added to the zinc, acid and water, and the hydrogen lighted, white fumes were emitted, with the characteristic odor of arsenious acid fumes, which, in opposition to authority, I will state is not the odor of garlic exactly, but nearer to that of phosphorus. On applying clear porcelain and glass to the flame, large metalic rings were obtained, so thick that they could readily be scaled off. These exhibited the characteristic metalic lustre and play of colors.

4th.—Some of the scales from the rings obtained by Marsh's process were heated in a tube resolved into arsenious acid, distilled water added, and boiled, and sulphureted hydrogen passed through the solution, which threw down the rich golden precipitate of sulphuret of arsenic or orpiment.

5th.—A solution obtained in the same manner as that which was treated with sulphureted hydrogen, was tested with ammonio-sulphate of copper, when a beautiful rich green precipitate of arsenite of copper was obtained.

6th.—A similar solution being tested with ammonio-nitrate of silver, gave the canary yellow precipitate of arsenite of silver, which changes to a greenish brown when exposed to the light for a short time.

7th.—Reinsch's test was used. Some of the decoction of coffee was acidulated with hydrochloric acid, boiled, and while boiling, bright slips of copper introduced into the boiling liquid, which were instantly covered with metallic arsenic.

One favorable circumstance in these cases was the large quantity of the poison administered, and on its being taken into the stomach with a quantity of food, the arsenic acted as an emetic in a few minutes, and most of it was ejected, most probably, with the food; but it is rare for so many cases to recover under the most favorable circumstances, and is, I think, an argument in proof of the potency as an antidote of the old remedy, magnesia, when persevered in, and given in large doses.—*Stethoscope.*

